# The Patellar Resurfacing in Total Knee Prosthesis: Indications for Bone Stock and Patellar Morphology

**DOI:** 10.3389/fmed.2020.00405

**Published:** 2021-02-24

**Authors:** Luigi Molfetta, Andrea Casabella, Augusto Palermo

**Affiliations:** ^1^DISC Department, Research Centre of Osteoporosis and Osteoarticular Disease, School of Medical and Pharmaceutical Sciences, University of Genoa, Genoa, Italy; ^2^Unit of Orthopaedic Surgery, IRCCS Istituto Auxologico Italiano, Capitanio Hospital, Milan, Italy

**Keywords:** knee, prosthesis, patellar resurfacing, anterior pain, revision, osteoporosis

## Abstract

The patellar resurfacing is still a controversial and unresolved problem. The choice to use the patellar resurfacing in the total knee prosthesis (TKP) is decided by the surgeon's experience; he analyzes the thickness, the shape, consumption of the surface and he chooses the use of patellar resurfacing or to limit itself to cheiloplasty, denervation, or often to the release of the lateral wing ligament. He also assesses the metabolic state of the bone linked to Osteoporosis and the potential fragility of the joint and kneecap in particular. Bone loss after total knee arthroplasty (TKP) may lead to periprosthetic fractures that are associated with significant costs (morbidity, economic, etc.) and pose a challenge to operative fixation. The literature doesn't express a definitive judgment on the two options, since the results can be overlapped on average. Each option has advantages and disadvantages to be considered in the overall balance of the patellar operation. In reality, however, this technical choice requires more consolidated decision-making criteria so as to minimize the incidence of post-surgical femoral-patellar pain syndrome, the second cause of failure, which frequently leads to revision of the implant. The balance between experience and evidence can be a compromise in the choice of surgery. The experience documented in the literature must identify the parameters capable of constructing an algorithm aimed not only at the secondary resurfacing rate, but at the overall clinical evaluation. This has implications also for the rehabilitation of these patients after surgery.

## Introduction

The management of the patella in total knee prosthesis (TKP) remains controversial. Some surgeons prefer the Patellar Resurfacing (PR) to counter the increase in revisions or other interventions and reduce the incidence of anterior knee pain such as in prosthesis without patellar resurfacing (NR). Other surgeons don't use the prosthesis to avoid complications such as fractures, tendon damage and secondary instability (1). In this procedural dichotomy we summarize the problem of the patella in the TKP. Since the 1980s, with the optimization of the design of the PTGs, the problem of the patella prosthesis has been posed, correlated with the incidence of postoperative anterior pain, which is not only patellar but multifactorial.

The average rates of PR vary in the various international cases; Fraser et al. have documented that in the period 2004–2014 the percentage of PR ranged from 4% (Norway) to 82% (United States), with Sweden between 15 and 2%, and Australia between % and 59% and with a global percentage value of 38% of PR in all registers outside the United States in 2010 (2).

Therefore, it is not a simple surgical option; it is based on the indication, on the technique, on the foreseeable outcome and today on the possible legal medical judgment.

The femoral-patellar pain in the TKP frequently leads to revision of the implant with the secondary patella operation, in variable percentage, up to 20%; it represents by implication the second cause of failure of the TKP (3, 4). Numerous randomized and controlled trials with a final end-point related to the clinical picture or to the prosthetic revision document this concept. Burnett et al. (5) in a randomized trial show the statistically significant equivalence between the two NR and PR groups about ROM, the clinical score, global and patella femoral pain and patient satisfaction; on the contrary, the revision rate (12%) in NR vs. (9%) R is higher. The same conclusions are reached by Ikejiani et al. (6) who in a retrospective study show that PR does not seem to influence clinical outcomes ROM, pain and postoperative complications. A dynamic-kinematic study conducted on NR and PR plants Pollo et al. (7) exclude statistically significant differences in groups about the biomechanics of walking, climbing stairs and getting up from the chair.

The surgeon doesn't use prosthesis of patella when it appears in good condition with not serious chondropathy, with good frontal alignment, of not excessive thickness, of good size, in right height, in young, not obese patient. Currently over 20 million people in the world have knee or hip joint arthroplasties, and unfortunately, there is a rise in periprosthetic fractures. The majority of these cases are fragility fractures which are difficult to manage surgically and are associated with high costs, prolonged length of stay, and poorer outcomes. Approximately one quarter of patients with osteoarthritis awaiting lower extremity arthroplasty have concomitant osteoporosis. Major risk factors for fracture are older age, female gender, and presence of osteoporosis which are common in those undergoing total knee arthroplasty (TKP). A potential strategy to reduce periprosthetic fracture risk is to identify suboptimal bone status and provide appropriate treatment if indicated. A frequently overlooked factor is the joint osteometabolic state and patella in particular. The flogosis present in the gonarthritis, especially in the most advanced states, characterizes not only the condropatia femoro-rotulea, it lacks the suffering of the subcondral bone that at RM is highlighted as Bone Marrow Lesion. In our experience, postoperative fragility fractures after TKA occur almost exclusively on the ipsilateral side. The etiology of these fractures is likely multifactorial (e.g., altered gait mechanics increasing ipsilateral falls); however, these events may be related to post-surgical weight-bearing changes through an implant leading to decreased ipsilateral BMD. Previous studies with small sample sizes have reported a decrease in ipsilateral distal femur BMD ranging from 1 to 44% (8, 9). However, there are no large studies that report results from multiple patient populations, surgeons, and implant designs. The verification of the good kneecap tracking and the absence of distal inpingment of the patella comfort the surgeon in the choice of non-prosthesis (10). Are these clinical elements sufficient to justify the choice of NR, or should we refer only to the percentage fear of secondary patellar resurfacing?

## Patellar Resurfacing vs. Not Resurfacing

The patellar resurfacing requires a preliminary evaluation of the functional anatomy of the knee in the prosthesis. In the clinical examination, femoral-patellar arthrosis has a different clinical profile for each patient for anterior pain intensity: pain-starter, difficulty in descending the stairs, hypo or immobility of the patella, size of the same compared to the lateral counter, etc. Preoperative pain, obesity or the degree of intraoperative chondromalacia have absolutely no predictive value of the possible anterior pain syndrome or a possible high need for secondary implant revision as pointed out by Barrach et al. (11) and by Marcacci et al. (12). From the clinical point of view, the presence of an evident painful anterior component of the degenerated knee (primitive or secondary) is a factor which generally suggests for PR. Radiographic examination of the patella in lateral and axial vision at 30° allows a more detailed morphological evaluation of thickness, height or osteophytosis. About the thickness of the patella, in cadaver studies, have shown as 5 mm thick plus 30% increase in contact stresses in knee flexion (13). The thicker patella limits the recovery of postoperative flexion and obliges the forced mobilizations for rigidity while the lateral osteophytosis influences the frontal kinematics of the quadricipital apparatus.

The CT better highlights the morphology of the patella and its relationships with the femur (14), just as the SPECT / CT method has been demonstrated in the study by Slevin et al. an ideal imaging modality for the evaluation of patellar-femoral disorders before and after TKP (15).

The thickness of the patella is the main element for the choice; 26 mm for men and 23 mm for women are the thicknesses, calibrated intraoperatively, of reference beyond which the surgeon must pose the problem, under which he can neglect it. According to Roessler et al. before the primary TKP, the inclination, the width and the thickness must be measured for the possible risk of post-surgical resurfacing (16). The thickness then correlates with the holding of the patella abutment resected for a possible secondary fracture. The thickness of the patella, although optimized, may not be sufficient if the femoral component is oversized and therefore thwarts the good technical gesture on the patella. Hsu et al. (17) confirm the need to reproduce the same pre-operative thickness in the PR; the decrease in thickness reduces the pressure, but increases the risk of fracture; an increase in thickness increases the low-bending quadricipital lever arm, but reduces the Range of Motion (ROM), as the compression forces increase 70 and 95° (18).

The position of the patella evaluated in the lateral radiograph according to various criteria, the main one of which the Insall-Salvati (19) correlates with the kinematics of the prosthesis itself; a low patella can create a distal impingement with component wear and a severe risk of separation. The correct femoral-patellar ratios provide that the patellar prosthesis should be placed on the resection area mainly in the upper middle and the more lateralized femoral component placed in external rotation, so as to ensure a more physiological patellar tracking. Therefore, it is necessary to avoid the flexion of the component, its anterior projection, the intrarotation or the medialization, negative factors for the kinematics and for the survival of the patellar component. Intrarotation is a negative factor of particular importance, well documented by Berger (20); the degree of rotation always greater, from 1 to 4°, 3 to 8°, or >8°, involves, respectively, a painful condition, the subluxation or the luxation with mobilization of the liner. The patellar prosthesis is therefore also influenced by the height of the articular line; a “high” rhyme due to excessive femoral resection creates greater ligament pararotulea tension and therefore pain; more important is a “low” patella for excessive tibial resection because it induces an impingement with the polyethylene insert. Figgie et al. establish the minimum limit of the joint interline in 8 mm which does not cause undesirable effects (21).

The deformation of the patella evaluated intraoperatively constitutes a further element of choice. The prosthesis becomes necessary when the shape of the patella is concave or flat; in this case the patellar tracking becomes impossible or in any case already predisposed to subluxation.

The degree of chondropathy according to Rodriguez-Merchan et al. obliges the patellar resurfacing; in grade IV, the revision rate of prosthesis in the study was 10 times greater (11.6%) than in the group with lower grade chondropathy (0.6%) (22). The same conclusion was reached by Schroeder-Boersch et al. (23) in a randomized 2-years study; the prosthesis in advanced patellar arthritis guarantees better functional results. The diagnosis of rheumatoid arthritis (RA), due to the peculiar condition of osteoporosis and of anatomical and pathological damage, makes the decision more problematic. Kawabubo et al. (24) analyzing the radiographic changes of the patella in the TKP of patients with RA, considers the mandatory resurfacing. On the contrary, the retrospective study by Seo et al. (25) shows instead that there is no correlation between the degree of articular defect and the patellar resurfacing in terms of clinical and radiological outcomes. Certainly the surgical gesture toward the anterior femoral patellar compartment is complex and also concerns the soft parts. Hwang et al. (26) have stated that the positive role of soft tissue balancing and prosthetic design can orientate toward a kneecap -plasty, even in the knees with severe f kneecap -femoral arthrosis. The patellar denervation with electrocautery was studied in a meta-analysis by Li T. et al. for the purpose of evaluating the reduction of postoperative pain in the anterior knee (Anterior knee pain = AKP). In the five randomized controlled trials (RCTs) with 572 patients and 657 knees, perirotuleal denervation was associated with improved pain and postoperative articular function, whereas complications did not differ significantly between the two groups (27). Denervation therefore supports the choices on resurfacing but is not a clear alternative to prosthetics.

The prosthetic design concerns the geometry and the shape of the components, especially of the femoral shield, in relation to the depth and width of the femoral throat and to the symmetry or not of the patellar pattern. Rader et al. have shown how the matel-backed of the patella component can affect the failure rate equal to 8.4% from 12 to 24 months, up to 33.3% after 6 years, with a 10-years projection of 50% of failure (28). Braakmann et al. analyzed the patella in poly, metal-backed and non-resurf and concluded that no difference exists in the three groups about ROM, stability, movement deficit, pain, alignment, walking distance and use of walking aids (29). Studies on total knee arthroplasty with LCS bearing have shown the importance of soft tissues, in particular ligaments, in the decision for patellar resurfacing or not (30).

## Patellar Resurfacing and Complications

The patellar resurfacing in PKT is inspired today, above all, by the surgeon's experience. Post-surgical anterior pain according to Burnet shows no statistically significant differences in PR and NR patients (5). Calvisi et al. in a study conducted on 5 meta-analyzes, a systematic review and six randomized trials, conclude that non-resurfacing leads to a higher incidence of pain, less surgical satisfaction, even if at the same recovery of the ROM (31). Breeman et al. in the largest randomized controlled trial of PR reported to date, the functional outcome, the rate of new intervention and the cost of total health care 5 years after total knee arthroplasty weren't significantly affected by the addition of resurfacing patellar to the surgical procedure (32). Functional results and subjective satisfaction are equivalent in the two strategies; however Barrack et al. they show how the possibility of secondary prosthesis due to post-prosthetic pain (11) increases in the NR patella. However, Bonnin et al. emphasizes that in the prosthetic patella but with insufficient or asymmetric bone resection, the revision percentage nevertheless reaches 5%; in this case the prosthesis is less complex and difficult than the revision of a patellar prosthetis (33). The surgeon who prosthesis the patella knows that he has to face his complications. The mobilization of the patellar prosthesis is generated by numerous causes: the metal back stimulates it up to 15% compared to polyethylene (4%), while the considerable thickness of the prosthetic patella increases the contact and cutting forces and therefore stimulates the separation (5). It should also be considered the metabolic state of the bone, as a pathogenic moment of cleavage for poor bone-prosthesis or bone-cement-prosthesis integration. Nonetheless, orthopedic surgeons, particularly those caring for patients with advanced knee OA, have shown relatively little interest in the management of osteoporosis. This may be due to the traditionally held belief that patients with advanced knee OA are less likely to develop osteoporosis. Several previous studies have reported the existence of an inverse relationship between osteoporosis and OA, particularly in the hip and knee Furthermore, a higher body mass index has been reported to increase the risks of the development and progression of OA of the knee but to decrease the risk of osteoporosis. Clements et al. (34) report the revision rate of 3.1% in NR and 5% in PR for 5 years. With reference to the anterior pain, the revision rate was 17% in NR and 1% in PR. Burnett et al. refer to differences between NR and PR that are not significant for ROM and patient satisfaction; for the anterior pain the difference between NR and PR was 12 and 3%, respectively; finally, the revision rate between NR and PR was 12 and 9%, respectively (35). The fracture of the patella, the most fearful complication, can occur intraoperatively or postoperatively with a stimulated frequency around 3%; it recognizes numerous pathogenic factors, such as the thickness of the patella due to excess of resection, osteoporosis, the technical gesture, revision, maltracking, denervation, and oateometabolic suffering related to the presence of district bone edema or the condition of general and joint osteoporosis. A considerable proportion of elderly female patients with advanced knee OA undergoing TKA also have osteoporosis. These anecdotal observations seemingly contradict the previously held inverse relationship between knee OA and osteoporosis. However, this inverse relationship had been demonstrated by the studies using community-based populations with various stages of OA and it is still unclear whether this relationship would also be found in patients with advanced knee OA undergoing TKA. If our observation is the case, more functional deterioration in the knee OA patients might be related to lower bone mineral density (BMD). However, little information is currently available regarding this speculation (36).

## Discussion

The analysis in Literature of the problem offers substantially overlapping results in the various systematic reviews, Cochrane or randomized studies. Both the PR and NR options can be used. Thus, post-prosthesis anterior pain and secondary resurfacing risk are the dominant elements of systematic analysis of knee prostheses. Each meta-analysis oscillates between the disadvantages of NR compared to the incidence of surgical revision and the disadvantages of PR over complications of kneecap prosthetics. There were no significant differences between PR and NR about the incidence of anterior knee pain; a higher rate of reoperations was observed in the NR group. The model of total knee replacement does not influence the incidence of secondary resurfacing; Pavlou et al. analyzed 18 randomized controlled trials of level I with a total sample size of 7,075 knees (3463 PR and 3612 NR), in order to reoperation rate, to anterior pain and to functional scores as outcome measures. The increased incidence of re-operations in the NR group should be considered simply as an additional surgical option for the treatment of anterior knee pain after TKP, thus artificially increasing the rate of re-interventions in the NR group (37). Longo et al. in the PR group they showed significantly higher postoperative pain with higher incidence of revision of the NR group (6.9 vs. 1%), concluding that primary resurfacing is the most effective option (38). Despite the same conclusions, Kai Chen et al. referring to a meta-analysis of 1,725 randomized trials, they consider the risk difference related to reoperation should be evaluated (39); Fu et al. (40) found that 76% of patients with postoperative anterior pain benefit from secondary resurfacing vs. a negative 24%. Post-prosthetic anterior pain is certainly a less parametric clinical parameter than the percentage statistic incidence of revisions. Fu et al. (40), He et al. (41), and Li et al. (42) they found no difference between the groups (PR and NR) in terms of anterior knee pain, but only in terms of increased risk of revision; Lindstrand et al. (43) documented the same incidence of revisions between the two groups. A prospective study by Patil et al. (44) related to three groups (PR, NR, and patelloflex) documented an improvement in the KSS scores and the patient satisfaction index, as well as a systematic review of 20 randomized controlled trials, whereby the reoperation rate of the patellar resurfacing group was lower than the non-resident group in the 1–2 year, follow-up; the differences disappeared and the incidences over 2 years equalized (45).

In conclusion, all the numerous papers analyzed state that studies designed with large samples and long-term follow-up are necessary, for a strong and definitive final recommendation about the kneecap resurfacing in the TKP. It is believed to share the conclusions of Grassi et al. (46): PR benefit is limited and it can reduce risks of secondary resurfacing. On a practical level, the results between PR and NR are comparable, as also supported by Pavlou et al. (37) who state that neither the PR nor the prosthetic design affect the clinical outcomes of TKP. Therefore, there is no clear superiority of R over NR (43). In a study of over 15,000 patients, the reasons for NR were the condition of the cartilage almost normal (56%), the young age (8%), the thin of patella (13%) and the choice of surgeons (23%) (47).

The increased risk of secondary resurfacing should be interpreted with caution due to the methodological limitations of the meta-analyzes concerning the search criteria, the heterogeneity and the intrinsic bias of an easier indication to reoperation when the kneecap was not prosthetic. Hans-Peter et al. (48) express the same judgment of caution about secondary resurfacing, considered a treatment option available to resolve post-prosthesis pain, even for non-homogeneous outcomes of secondary resurfacing itself; the Authors believe that there is currently no evidence-based recommendation for the use of secondary kneecap resurfacing; it would be desirable according to van Jonbergen et al. (48) a uniformly validated scoring system to analyze retro kneecap degeneration for secondary resurfacing purposes. Finally, the authors point out that the cause of anterior knee pain after TKP is not always related to the patella, but to other causes such as the insufficient posterior cruciate ligament, the intrarotiated femoral and/or tibial component, the tendinosis, etc. Therefore, secondary resurfacing is a still controversial procedure with uncertain results and burdened by complications (infections and alterations of wound healing, patellar instability and patellar fracture). This systematic review supports only a weak recommendation for secondary patellar resurfacing if patient satisfaction and clinically important improvement of functional outcomes are the desired endpoints.

Koh et al. (49) underline that the alleged improvement of secondary PR produces a satisfactory result in two out of five operated patients, who were oblivious to the advantages of the second surgical treatment, implemented to eliminate the anterior pain (50).

In conclusion, protected by evidence based medicine that considers both surgical options (PR and NR) both the multifactorial pain in NR or the need for secondary R cannot be considered to be a weak choice or a malpractice error. The role of the surgeon's experience emerges and of his ability to evaluate all the parameters in a clinical reasoning that analyzes as many factors as possible to prosthetize or not the patella, in the absence of clear and definitive recommendations of the Literature. In addition to secondary effect of TKP, number of previous studies in community-based populations have presented evidence of an inverse relationship between osteoporosis and OA. However, little information is available on whether patients with advance knee OA would be far less likely to develop osteoporosis by the inverse relationship. As the patients with advanced knee OA have elevated risks of incident vertebral and non-vertebral (including hip) fractures, precise information on the nature of BMD in this patient group would be valuable to those involved in patient care and to those interested in social health care burden imposed by advanced knee OA.; and so advanced knee OA *per se* does not have a marked protective effect against osteoporosis demostring that that more attention should be paid to identification and treatment of osteoporosis in elderly female patients with advanced knee OA undergoing TKP.

## Author Contributions

All authors listed have made a substantial, direct and intellectual contribution to the work, and approved it for publication.

## Conflict of Interest

The authors declare that the research was conducted in the absence of any commercial or financial relationships that could be construed as a potential conflict of interest. The reviewer RB declared a shared affiliation, though no other collaboration, with one of the authors LM to the handling editor.
